# Supplementary material for: Hybrid Models and Biological Model Reduction with PyDSTool
Source: PLoS Comput Biol. 2012 Aug 9;8(8):e1002628. doi: 10.1371/journal.pcbi.1002628 (PMC3415397; doi:10.1371/journal.pcbi.1002628)
Supplement: Text S4 — Complete source code for the PyDSTool package (version 0.88.120504). Includes API documentation and help files linking to web pages. This file is identical to the current public release on Sourceforge.net. (ZIP) [file pcbi.1002628.s004.zip › PyDSTool/html/PyDSTool.Symbolic.QuantSpec-class.html]

xml version="1.0" encoding="ascii"?


PyDSTool.Symbolic.QuantSpec


| Home | Trees | Indices | Help | PyDSTool |
| --- | --- | --- | --- | --- |

Package PyDSTool ::
Module Symbolic ::
Class QuantSpec

[hide private]

[frames] | no frames]

# Class QuantSpec

source code

```
object --+
         |
        QuantSpec
```

---

Specification for a symbolic numerical quantity.


|  |  |
| --- | --- |
| Instance Methods | [hide private] |

|  |  |
| --- | --- |
| \_\_init\_\_(self, subjectToken, specStr=`'``'`, specType=None, includeProtected=True, treatMultiRefs=True, preserveSpace=False, ignoreSpecial=None)  x.\_\_init\_\_(...) initializes x; see x.\_\_class\_\_.\_\_doc\_\_ for signature | source code |

|  |  |
| --- | --- |
| \_check\_self\_ref(self) | source code |

|  |  |
| --- | --- |
| redefine(self, specStr) | source code |

|  |  |
| --- | --- |
| isspecdefined(self) | source code |

|  |  |
| --- | --- |
| isDefined(self, verbose=False) | source code |

|  |  |
| --- | --- |
| \_\_call\_\_(self, \*args)  str(x) | source code |

|  |  |
| --- | --- |
| isCompound(self, ops=`[``'``+``'``,` `'``-``'``,` `'``*``'``,` `'``/``'``]`) | source code |

|  |  |
| --- | --- |
| \_\_contains\_\_(self, tok) | source code |

|  |  |
| --- | --- |
| \_\_getitem\_\_(self, ix) | source code |

|  |  |
| --- | --- |
| \_\_setitem\_\_(self, ix, s) | source code |

|  |  |
| --- | --- |
| \_\_delitem\_\_(self, ix) | source code |

|  |  |
| --- | --- |
| \_\_combine(self, other, opstr, reverseorder=0) | source code |

|  |  |
| --- | --- |
| \_\_add\_\_(self, other) | source code |

|  |  |
| --- | --- |
| \_\_mul\_\_(self, other) | source code |

|  |  |
| --- | --- |
| \_\_sub\_\_(self, other) | source code |

|  |  |
| --- | --- |
| \_\_div\_\_(self, other) | source code |

|  |  |
| --- | --- |
| \_\_radd\_\_(self, other) | source code |

|  |  |
| --- | --- |
| \_\_rmul\_\_(self, other) | source code |

|  |  |
| --- | --- |
| \_\_rsub\_\_(self, other) | source code |

|  |  |
| --- | --- |
| \_\_rdiv\_\_(self, other) | source code |

|  |  |
| --- | --- |
| \_\_truediv\_\_(self, other) | source code |

|  |  |
| --- | --- |
| \_\_rtruediv\_\_(self, other) | source code |

|  |  |
| --- | --- |
| \_\_rpow\_\_(self, other) | source code |

|  |  |
| --- | --- |
| \_\_pow\_\_(self, other, reverseorder=0) | source code |

|  |  |
| --- | --- |
| \_\_neg\_\_(self) | source code |

|  |  |
| --- | --- |
| \_\_pos\_\_(self) | source code |

|  |  |
| --- | --- |
| \_\_len\_\_(self) | source code |

|  |  |
| --- | --- |
| \_\_repr\_\_(self)  repr(x) | source code |

|  |  |
| --- | --- |
| \_\_str\_\_(self, \*args)  str(x) | source code |

|  |  |
| --- | --- |
| \_\_eq\_\_(self, other, diff=False) | source code |

|  |  |
| --- | --- |
| \_\_ne\_\_(self, other) | source code |

|  |  |
| --- | --- |
| difference(self, other)  Show differences in comparison to another QuantSpec. | source code |

|  |  |
| --- | --- |
| isvector(self)  Is the quantity spec of the form [a,b,c] | source code |

|  |  |
| --- | --- |
| fromvector(self, ix=None)  Turn the QuantSpec's specification of a vector to a list of the element's specifications. | source code |

|  |  |
| --- | --- |
| simplify(self)  Simplify expression \*IN PLACE\*, but do not substitute terms from other quantities or reduce constants such as Pi to a floating point number. | source code |

|  |  |
| --- | --- |
| renderForCode(self)  Return code-ready version of spec (e.g. | source code |

|  |  |
| --- | --- |
| eval(self, \*scopearg, \*\*defs)  Evaluate expression, in an optional scope dictionary (defaults to locals). | source code |

|  |  |
| --- | --- |
| \_eval(self, eval\_type, \*scopearg, \*\*defs)  Internal helper function for eval. | source code |

|  |  |
| --- | --- |
| tonumeric(self) | source code |

|  |  |
| --- | --- |
| mapNames(self, nameMap)  Can pass a dictionary or a symbolMapClass instance to this method, but it will convert it internally to a symbolMapClass. | source code |

|  |  |
| --- | --- |
| \_\_copy\_\_(self) | source code |

|  |  |
| --- | --- |
| \_\_deepcopy\_\_(self, memo=None, \_nil=`[``]`) | source code |

**Inherited from `object`**:
`__delattr__`,
`__getattribute__`,
`__hash__`,
`__new__`,
`__reduce__`,
`__reduce_ex__`,
`__setattr__`


|  |  |
| --- | --- |
| Properties | [hide private] |

**Inherited from `object`**:
`__class__`


|  |  |
| --- | --- |
| Method Details | [hide private] |

|  |  |
| --- | --- |
| \_\_init\_\_(self, subjectToken, specStr=`'``'`, specType=None, includeProtected=True, treatMultiRefs=True, preserveSpace=False, ignoreSpecial=None)  *(Constructor)* | source code |

x.\_\_init\_\_(...) initializes x; see x.\_\_class\_\_.\_\_doc\_\_ for
signature

Overrides: object.\_\_init\_\_: *(inherited documentation)*

|  |  |
| --- | --- |
| \_\_repr\_\_(self)  *(Representation operator)* | source code |

repr(x)

Overrides: object.\_\_repr\_\_: *(inherited documentation)*

|  |  |
| --- | --- |
| \_\_str\_\_(self, \*args)  *(Informal representation operator)* | source code |

str(x)

Overrides: object.\_\_str\_\_: *(inherited documentation)*

|  |  |
| --- | --- |
| renderForCode(self) | source code |

Return code-ready version of spec (e.g. uncapitalize built-in function
names)

|  |  |
| --- | --- |
| eval(self, \*scopearg, \*\*defs) | source code |

Evaluate expression, in an optional scope dictionary (defaults to
locals). Explicit definitions override scope dictionary definitions.

Scope dictionary maps variable name -> defining object.

|  |  |
| --- | --- |
| \_eval(self, eval\_type, \*scopearg, \*\*defs) | source code |

Internal helper function for eval. The use of indirection using \_eval
actually allows the correct scope to be accessed by whoQ when this
function automatically collects definitions.

  


| Home | Trees | Indices | Help | PyDSTool |
| --- | --- | --- | --- | --- |

|  |  |
| --- | --- |
| Generated by Epydoc 3.0.1 on Fri May 4 15:24:07 2012 | http://epydoc.sourceforge.net |
